# The diagnostic value of metagenomic next-generation sequencing versus traditional microbiological testing in native pyogenic spinal infections: A systematic review and meta-analysis

**DOI:** 10.1016/j.xnsj.2025.100840

**Published:** 2025-12-20

**Authors:** Othman Ibrahim, Rewa Aboushaala, Namrah Ahmed, Andrew Savoia, Sloane O. Ward, Shriya N. Patel, Gregory Lopez, Sarah E. Sansom, Brett Williams, Kern Singh, Lena Al-Harthi, Khaled Aboushaala

**Affiliations:** a Department of Microbial Pathogens and Immunity, Rush Medical College, Rush University Medical Center, 1735 W. Harrison St. Cohn Bldg, Chicago, IL, 60612, United States; b Department of Orthopedic Surgery, Rush Medical College, Rush University Medical Center, 1611 W. Harrison St.Chicago, Chicago, IL, 60612, United States; c Department of Internal Medicine, Division of Infectious Diseases, Division of Clinical Microbiology, Rush Medical College, Rush University Medical Center, Chicago, 600 S Paulina St #140, Chicago, IL, 60612, United States

**Keywords:** Metagenomic next-generation sequencing, mNGS, Spinal infections, Diagnostic accuracy, Pathogen detection, Culture-negative infections, Microbial identification, Vertebral osteomyelitis, Spondylodiscitis, Spinal epidural abscess

## Abstract

**Background:**

Native pyogenic spinal infections (PSIs), including spondylodiscitis and vertebral osteomyelitis, are challenging to diagnose due to low culture sensitivity and delayed results. Metagenomic next-generation sequencing (mNGS) has emerged as a promising diagnostic tool, but its comparative clinical utility remains uncertain. The purpose of this study is to systematically compare the diagnostic performance and clinical impact of mNGS versus conventional microbial culture in detecting pathogens responsible for native PSIs.

**Methods:**

The current systematic review and meta-analysis was conducted in accordance with Preferred Reporting Items for Systematic Reviews and Meta-Analyses (PRISMA) guidelines. A comprehensive literature search was performed across 6 major databases. Eligible studies directly compared mNGS with standard culture for native PSIs and reported diagnostic performance metrics. Data were extracted and analyzed using a random-effects model to produce pooled estimates. Study quality was assessed using the Newcastle-Ottawa Scale. Primary outcomes included pooled sensitivity, specificity, positive predictive value (PPV), and negative predictive value (NPV). Secondary outcomes assessed diagnostic yield, time to diagnosis, treatment modification, and false-positive or contamination events.

**Results:**

A total of 1,227 patients from 12 studies were included, encompassing those with suspected or confirmed native PSIs. Pooled sensitivity and specificity of mNGS were 89.7% (95% CI: 85.6–93.1%) and 86.2% (95% CI: 80.5–91.0%), respectively. mNGS demonstrated a significantly higher diagnostic yield (69–90%) compared to culture (27.2–44.7%) and enabled faster diagnosis (range, 17.7–48 hours). mNGS informed antimicrobial selection in up to 70.3% of cases and detected a broader pathogen spectrum. The incidence of false positives was low (range, 1–5) but non-negligible, emphasizing the need for careful interpretation.

**Conclusions:**

mNGS outperforms conventional culture in sensitivity, speed, and breadth of pathogen detection in native PSIs and supports more tailored antimicrobial therapy. However, careful interpretation is necessary due to potential false positives. These findings support the integration of mNGS into clinical workflows, particularly in complex or culture-negative infections.

## Introduction

Native pyogenic spinal infections (PSIs), which includes conditions such as pyogenic spondylodiscitis, vertebral osteomyelitis, and spinal epidural abscesses, are serious and potentially life-threatening infections [1], associated with considerable morbidity and mortality. Timely diagnosis and correct identification of the etiologic microorganisms are critical to inform appropriate antimicrobial therapy, reduce improper use of broad-spectrum antibiotics, and enhance short- and long-term clinical outcomes [2]. Although culture-based methods, such as blood cultures, image-guided needle spinal aspiration, and open biopsy are well-established for PSI diagnosis, they have substantial limitations.

Over the past decade, metagenomic next-generation sequencing (mNGS) has emerged as a powerful diagnostic tool [3], which enables identification of a broad spectrum of pathogens, such as bacteria, viruses, fungi, and parasites-from clinical specimens such as tissue, pus, or blood [4]. mNGS can identify rare, fastidious, or unusual pathogens commonly missed using routine cultured-based methods. The rising burden of spinal infections and limitations of conventional diagnostics highlight the need for a focused evidence synthesis [5]. A systematic comparison of mNGS and culture methods can clarify their diagnostic performance and clinical utility. Data on how often mNGS influences treatment decisions, reduces time to diagnosis, or improves outcomes remain largely unquantified [6], yet are essential for developing evidence-based guidelines.

Key research gaps include the limited evaluation of mNGS diagnostic performance in native PSIs and its influence on clinical decision-making. Data are scarce on unique pathogen detection by mNGS and how often these findings alter treatment. Concerns about false positives and contamination led to a systematic review and meta-analysis comparing mNGS with conventional culture [7]. The current analysis aimed at estimating and comparing pooled sensitivity, specificity, and diagnostic yield measures between the 2 techniques. Secondary objectives were formulated to assess the broader clinical utility of mNGS, which included evaluating the frequency with which mNGS results had led to changes in antimicrobial therapy, comparing the average time to pathogen identification between mNGS and traditional culture and determining the incidence and clinical impact of false-positive findings or contamination.

## Materials and methods

### Study design and research questions

This systematic review was performed following the Preferred Reporting Items for Systematic Reviews and Meta-Analyses (PRISMA) 2020 guidelines. The protocol was registered in PROSPERO under number: CRD420251041667. Table 1 shows the PICO statement of the current study. This review pooled data from randomized controlled trials, observational cohort studies, pilot studies, and case series directly comparing the diagnostic yields of mNGS vs. culture-based testing in the setting of native PSIs.

**Table 1:** PICO statement.

Element	Description
Population (P)	Patients with native pyogenic spinal infections (eg, spondylodiscitis, vertebral osteomyelitis, spinal epidural abscesses)
Intervention (I)	Pathogen identification using mNGS
Comparator (C)	Conventional microbiological diagnostics (primarily culture-based methods)
Outcomes (O)	Diagnostic yield, sensitivity, specificity, PPV, NPV, time to diagnosis, changes in antimicrobial therapy, detection of additional pathogens, false positives or contamination

Abbreviations: mNGS, metagenomic next-generation sequencing; PPV, positive predictive value; NPV, negative predictive value.

To guide the systematic review, 5 research questions were formulated. First, what had been the pooled sensitivity and specificity of mNGS compared with traditional culture for diagnosing native PSIs? Second, had mNGS provided a higher diagnostic yield in patients whose infections had remained culture-negative? Third, has mNGS reduced the time required for pathogen identification in comparison to traditional microbiology? Fourth, what proportion of pathogens had been detected exclusively by mNGS and not by conventional methods, and what had been the clinical relevance of these findings? Fifth, what risks, and harms had previously been linked to false-positive or contaminant detection by mNGS?

### Study selection, data extraction, quality assessment, and meta-analysis

Studies were included based on the defined inclusion and exclusion criteria (Table 2). An extensive literature search was performed using the following electronic databases: PubMed, EMBASE, Scopus, Web of Science, Cochrane Library, and MEDLINE. Gray literature was also searched using Google Scholar and OpenGrey to find relevant unpublished or nonindexed studies. Table 3 shows the keyword strings used in the search. Two independent reviewers screened the titles and abstracts against the inclusion and exclusion criteria. Full-text versions of potentially eligible studies were assessed for final inclusion.

**Table 2:** Inclusion and exclusion criteria of the studies.

Characteristics	Inclusion criteria	Exclusion criteria
Study type	Randomized controlled trials, prospective or retrospective cohort studies, pilot studies, and case series	Case reports, conference abstracts without peer-reviewed full texts, editorial comments, narrative or systematic reviews
Study objective	mNGS contrasted with standard culture techniques in diagnosing native spinal infections	Studies which did not report comparative data for mNGS and standard culture or did not have extractable outcome data
Study outcomes	Sensitivity, specificity, PPV, NPV, treatment modification, diagnostic yield, and time to pathogen identification	Incomplete data
Other criteria	Full-text articles published in the English language	No full text; published in languages other than English.

Abbreviations: mNGS, metagenomic next-generation sequencing; PPV, positive predictive value; NPV, negative predictive value.

**Table 3:** Keyword strings used in the search.

Strings used	((“spinal infection" OR "spondylodiscitis" OR "vertebral osteomyelitis") AND ("metagenomic sequencing" OR "next-generation sequencing" OR "mNGS")) AND (("diagnosis" OR "pathogen detection" OR "microbial identification"))

Any discrepancies between reviewers were resolved through consensus or adjudication by a third reviewer. From each eligible study, the data were systematically extracted using a standardized form aligned with the study objectives (Table 4). The quality of the included studies evaluating the diagnostic performance of mNGS in spinal infections was assessed using the Newcastle-Ottawa Scale (NOS) [8], a validated tool for evaluating nonrandomized studies. A reliable manual approach using a random-effects model (DerSimonian–Laird method) was employed to generate forest and funnel plots using R Statistical Software (v4.1.2).

**Table 4:** Data extracted from the studies.

Category	Parameters to be analyzed
1. Study characteristics	• Study number
	• Authors and publication year
	• Study type (RCT, cohort, case series, pilot, retrospective, prospective, etc.)
2. Population demographics	• Number of participants
	• Gender distribution (male/female)
	• Age (mean, median, or range)
	• Underlying clinical conditions
3. diagnostic performance metrics	• Sensitivity of mNGS
	• Specificity of mNGS
	• PPV
	• NPV
4. CLINICAL utility outcomes	• Diagnostic yield (% positive pathogen identification by mNGS)
	• Time to diagnosis (hours or days)
	• Type and frequency of treatment modification informed by mNGS (escalation, de-escalation, substitution)
	• Antimicrobial management changes guided by mNGS
	• Adverse diagnostic outcomes (eg, misdiagnosis, unnecessary therapy)
	• False positives or contamination events (n, %)
	• Additional pathogens detected by mNGS but missed by culture
5. Study limitations and author conclusions	• Reported study limitations
	• Main conclusion/clinical relevance

Abbreviations: mNGS, metagenomic next-generation sequencing; RCT, randomized controlled trial; PPV, positive predictive value; NPV, negative predictive value.

## Results

**Fig. 1:**
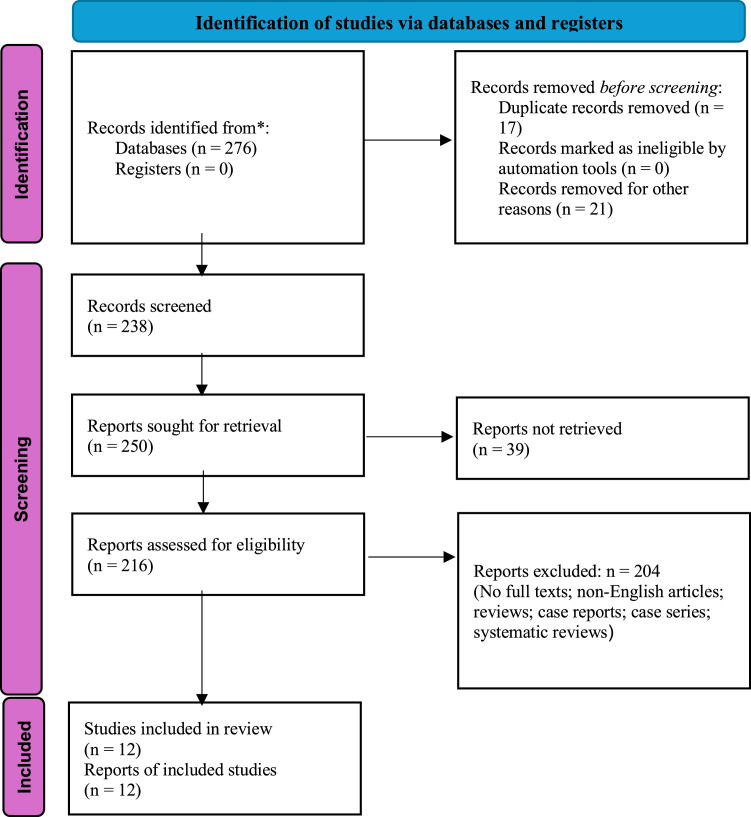
PRISMA flow chart.

Fig. 1 shows the completed PRISMA flow chart demonstrating the screening process of the study. A total of 12 studies involving 1,227 patients were included in this meta-analysis and systematic review, evaluating diagnostic performance and clinical utility of mNGS for spinal infections. Study designs were retrospective (7 studies) and prospective (5 studies) observational cohorts, between 2022 and 2025. Most participants had confirmed or suspected spinal infection, with subgroups being neuroborreliosis, PSI, spinal tuberculosis (STB), and mixed microbial aetiologies. Supplementary Table 1 shows the complete data extraction of the study.

NOS scores are shown in Table 5. Four studies [[9], [10], [11], [12]] had the maximum score of 9 on the NOS, reflecting robust study design, clearly defined participant selection, sufficient comparability, and unambiguous outcome reporting. Table 6 shows the demographic characteristics of the patients in the included studies and Table 7 shows the risk of bias assessment of the studies.

**Table 5:** Newcastle-Ottawa scale domains and scores.

Domain	Criteria assessed	Maximum points
Selection	− How participants were recruited − Representativeness of cohorts − Appropriateness of cohort definition	4
Comparability	Control for confounding variables (eg, age, gender, disease severity)	2
Outcome	− Clarity and validity of outcome measurement − Reliability of diagnostic methods − Completeness of outcome reporting	3
Total score	Sum of scores from all domains	9

**Table 6:** Demographic characteristics of the patients in the included studies.

Authors	Participants (n)	Male (n)	Female (n)	Age (years)	Clinical conditions
Li et al. [12]	20	13	7	Median 42 (IQR: 30–52)	Neurobrucellosis (NB)
Yin et al. [15]	120	80	40	Inf: 66.0±11.8; Noninf: 59.2±12.4	Spinal infections vs noninfectious spinal diseases
Wang et al. [8]	114	61	53	Mean 62±11.7 (infection group)	Acute spinal infections & controls
Li et al. [14]	301	186	115	Mean 61.6±13.4	Pyogenic spinal infections
Zhang et al. [13]	38	29	9	Mean 57.4±12.9	Spinal infections (vertebral osteomyelitis, discitis, abscesses, etc.)
Li et al. [9]	27	12	15	Median 61.5 (Range: 40–83)	Tuberculous spondylitis, fungal, *Brucella*, viral, mixed
Xu et al. [17]	108	55	53	Median 57.8 (Range: 14–82)	Spinal infections (lumbar, thoracic, cervical)
Li et al. [18]	126	76	50	STB: 52.6±18.8; non-STB: 53.9±15.3	Suspected spinal infections (STB & non-STB)
Li et al. [10]	100	52	48	Mean 47.7±16.5	Spinal TB, non-TB spinal infection, noninfectious cases
Ali et al. [16]	85	49	36	Mean 54.5±19.2	CHD, DM, COPD with infections
Zhang et al. [11]	158	81	77	Mean 54	Spinal infections
Ma et al. [19]	30	16	14	Mean 63.0±9.0	Suspected spinal infections (infected: 26, aseptic: 4)

Abbreviations: IQR, interquartile range; CHD, chronic heart disease; DM, diabetes mellitus; COPD, chronic obstructive pulmonary disease; TB, tuberculosis.

**Table 7:** Risk of bias assessment of the included studies.

Author	Selection (4)	Comparability (2)	Outcome (3)	Newcastle-Ottawa scale score (out of 9)	Study quality
Li et al. [12]	3	1	2	6	Moderate
Yin et al. [15]	3	1	2	6	Moderate
Wang et al. [8]	4	2	3	9	High
Li et al. [14]	3	2	2	7	Moderate
Zhang et al. [13]	3	1	2	6	Moderate
Li et al. [9]	4	2	3	9	High
Xu et al. [17]	3	1	2	6	Moderate
Li et al. [18]	3	1	2	6	Moderate
Li et al. [10]	4	2	3	9	High
Ali et al. [16]	3	1	2	6	Moderate
Zhang et al. [11]	4	2	3	9	High
Ma et al. [19]	3	1	2	6	Moderate

### Diagnostic performance of mNGS

The diagnostic efficacy of mNGS showed persistently high performance in all studies included, albeit with variability in individual outcomes. Sensitivity was between 39.0% and 97.83%, with greater values seen more commonly in acute spinal infection and STB cases. Specificity had wider variability, between 16.2% and 100%, with lower values most often noted in those including control groups of chronic noninfectious spinal disorders. While PPV and NPV were not uniformly reported, data that were available suggested a high PPV—up to 100% (85.29– 100%), and more variable NPV, 16.2% to 96.9%. The meta-analysis provided a pooled sensitivity of 89.7% (95% CI: 85.6–93.1%) and a pooled specificity of 86.2% (95% CI: 80.5–91.0%).

### Diagnostic yield and time to diagnosis

Diagnostic yield of mNGS varied between 69% and 90%, whereas standard culture methods isolated pathogens in only 27.2% to 44.7% of cases. In settings with optimized protocols, such as obtaining samples before initiating antibiotics, culture yields may exceed 75%. Significantly, mNGS was capable of detecting organisms in culture-negative samples in all the included studies, with 1 study crediting 73.1% of its total detections to mNGS alone. Apart from higher yield, mNGS also exhibited a much shorter turnaround time. Diagnostic results were reported by most studies within 24 to 48 hours with mNGS, compared to the 2 to 7 days usually taken for culture-based techniques.

### Impact on antimicrobial management

Wang et al. [9] presented that 41.46% of patients' antibiotic regimens were modified based on mNGS results, often switching from empirical broad-spectrum therapy to targeted pathogen treatment. Similarly, Zhang et al. [12] identified that 80 patients (50.6%) underwent tailored antimicrobial treatment guided by mNGS information. These treatment adjustments included the introduction of antifungal therapy like voriconazole, the addition of antitubercular therapy, or the administration of doxycycline for atypical bacterial infection.

### False positives and contamination

Among the 12 included studies, Li et al. [13], Zhang et al. [14], and Li et al. [13] used a panel of infectious disease (ID) experts to adjudicate whether organisms were clinically significant or contaminants. Li et al. [15] and Li et al. [11] relied on clinical correlation and imaging as part of the adjudication process. Yin et al. [16], Wang et al. [9], and Ali et al. [17] used composite reference standards, including surgical findings, histopathology, and treatment response. Xu et al. [18], Li et al. [19], and Zhang et al. [12] did not clearly report their adjudication method. Incidents of contamination or false positives were generally low. One potential false positive was reported in Wang et al. [9]. Rare detection of potential contaminants, such as *Taifanglania major*, was noted in Xu et al. [18]. Background skin flora, including *Propionibacterium acnes*, were detected in Yin et al. [16], Li et al. [15], and Zhang et al. [14], though not always classified as false positives.

Li et al. [13] reported 5 discordant results between mNGS and conventional microbiological tests (CMTs); however, the exact incidence of false positives was not given. No false positives were reported in Li et al. [11]. Contamination in conventional cultures (not mNGS) was noted in Ali et al. [17] and around 25% of aseptic cases showed contamination in the study by Ma et al. [20]. No reporting on false positives or contaminants was found in Li et al. [13], Li et al. [13], and Zhang et al. [12].

### Pathogen detection beyond culture

mNGS detected a more diverse range of pathogens compared to conventional culture methods, identifying over 15 distinct pathogens missed by culture. These included unusual and fastidious organisms such as *Brucella, Coxiella burnetii, Mycoplasma hominis, Taifanglania major, Staphylococcus aureus, Escherichia coli, Klebsiella variicola*, various *Candida* species, *Aspergillus, Streptococcus agalactiae, Streptococcus anginosus, Klebsiella pneumoniae, Pseudomonas aeruginosa, Mycobacterium tuberculosis* complex, and multiple nontuberculous mycobacteria (NTM), fungi, and viruses that are often undetectable using conventional culture techniques. These results highlight mNGS's capability to identify a broad spectrum of infectious agents, such as those that would otherwise go undiagnosed with conventional culture-based approaches.

### Meta-analysis – sensitivity and specificity analyses

**Fig. 2:**
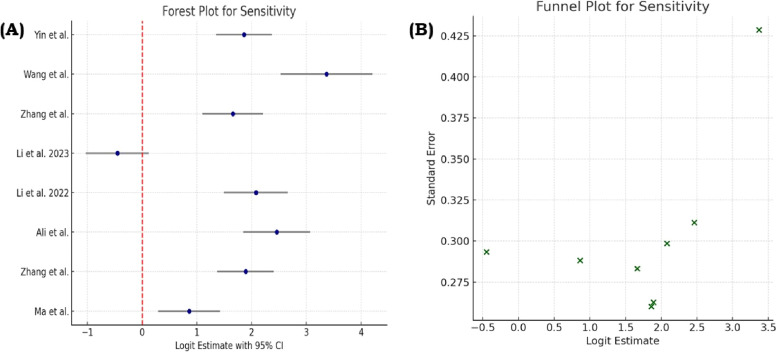
(A) Forest plot showing the pooled sensitivity estimates from included studies, with each study’s point estimate and 95% confidence interval displayed to illustrate between-study variability. (B) Funnel plot assessing potential publication bias for sensitivity, where the distribution of studies around the summary estimate is examined for asymmetry.

**Fig. 3:**
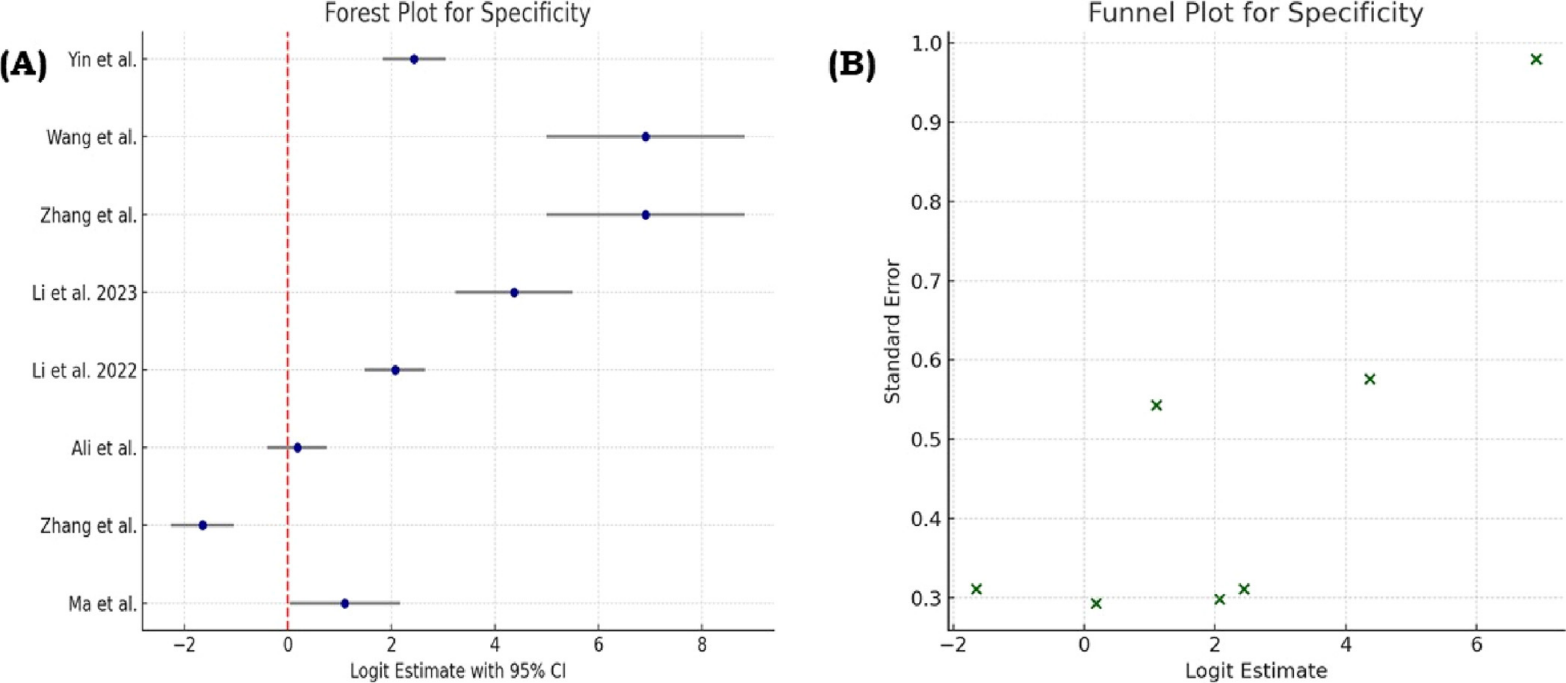
(A) Forest plot showing the pooled specificity estimates from included studies, with individual study effect sizes and corresponding 95% confidence intervals. (B) Funnel plot evaluating publication bias for specificity by visualizing the dispersion and symmetry of study results.

**Fig. 4:**
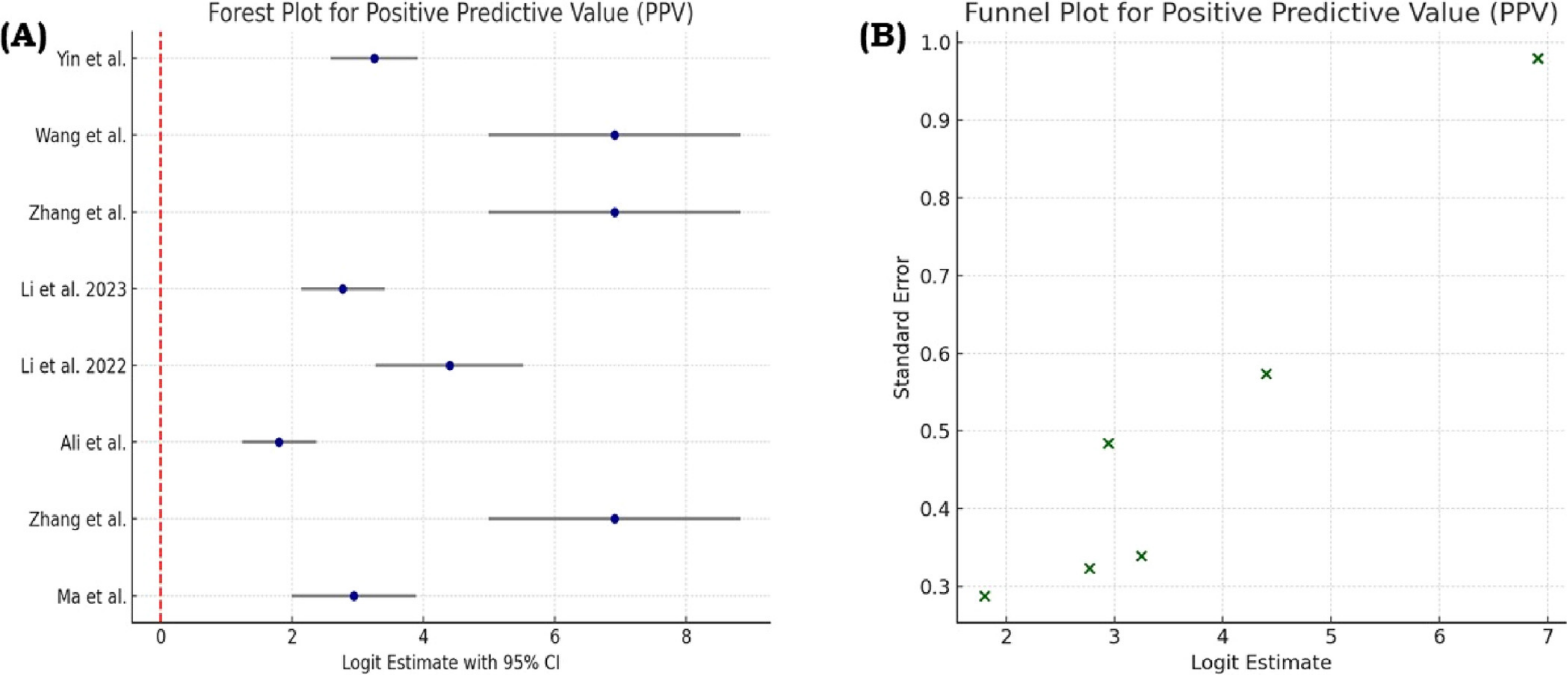
(A) Forest plot presenting the pooled positive predictive value estimates for all eligible studies, including the point estimates and 95% confidence intervals. (B) Funnel plot examining potential publication bias in PPV results through assessment of study distribution and symmetry relative to the pooled effect.

**Fig. 5:**
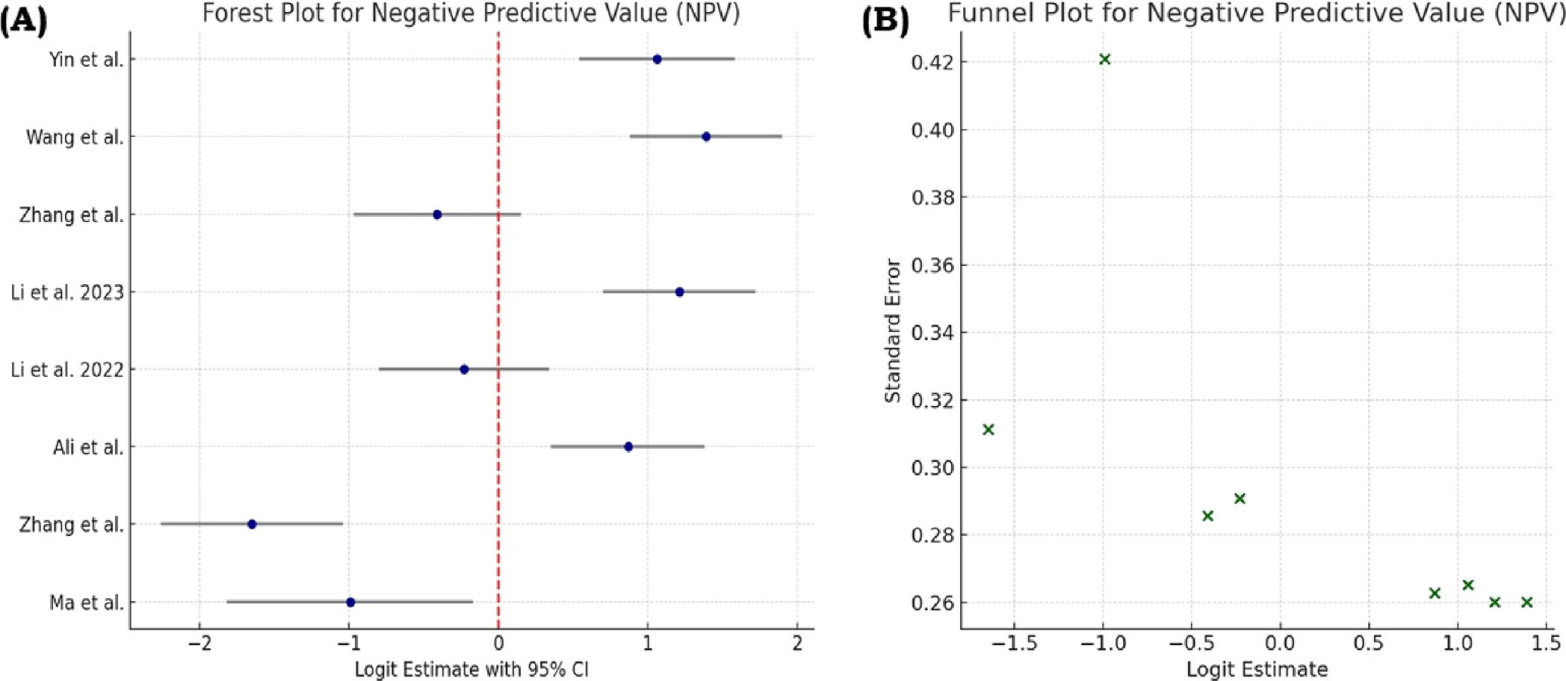
(A) Forest plot showing the pooled negative predictive value estimates across the included studies, displaying individual study estimates with their 95% confidence intervals. (B) Funnel plot assessing publication bias for NPV by visual inspection of the spread and symmetry of study findings.

We included 7 studies [16,9,14,19,11,17,12], Ma et al. (2022) in the meta-analysis, which reported all 4 diagnostic performance metrics (sensitivity, specificity, PPV, and NPV). Four studies [13,15,10,18] were excluded due to missing values in 1 or more required fields. Sensitivity estimates ranged broadly across studies, with values ranging from 39% to 89%, while specificity estimates varied from 16.2% to 88.9%. Similarly, PPV and NPV showed considerable variation between studies. Forest plots depicting pooled estimates and funnel plots assessing the sensitivity, specificity, PPV, and NPV are shown in Figs. 2, 3, Fig. 4, Fig. 5, respectively.

## Discussion

This systematic review and meta-analysis show that mNGS may provide a highly sensitive, clinically useful diagnostic method for spinal infections. In our analysis, mNGS demonstrated advantages over conventional culture methods by improving diagnostic yield, reducing time to diagnosis, and enhancing pathogen-specific treatment decisions. Similarly, previous findings by Zhang et al. [21] and Rajendiran et al. [22] analyzed the traditional and mNGS approaches to detect pathogenic organisms causing sepsis and reported that mNGS is superior to traditional culture methods. The study has also added that mNGS sensitivity is not affected by the infection site or sample types.

A retrospective cohort study on patients with pyogenic vertebral osteomyelitis reported that higher C-reactive protein levels (aOR 1.18) and open surgical biopsy (aOR 6.33) were significantly associated with culture positivity [23]. In the current analysis, considering the complex microbial background of spinal tissues, specificity stayed within a reasonable range even if it differs between studies. Traditional cultural methods sometimes fail in cases of past antibiotic exposure or fastidious organisms [24], and it may often fail to detect certain pathogens, as seen in studies where mNGS successfully identified *Brucella* [13,14], *Coxiella burnetiid* [16], and various mycobacteria, including NTM [18]. Similarly, in our analysis, we found that mNGS overcomes the limitations of conventional diagnostics by directly identifying nucleic acids independent of viability, thereby increasing diagnostic confidence in complex clinical cases. Additionally, mNGS enabled the identification of *Mycoplasma hominis* [12], further highlighting its role in improving pathogen detection beyond conventional culture-based methods.

Because spinal infections can rapidly lead cause neurologic compromise, their management mostly depends on time-to-result. Ma et al. [20] reported similar findings, stating that mNGS identified spinal infections in over 70% of patients, outperforming conventional culture-based methods. The 2 most commonly detected organisms in this study were *Staphylococcus* species and *Mycobacterium tuberculosis*. Similarly, a study by Zhang et al. [25] mNGS demonstrated a significantly higher detection rate than culture (74.4% vs. 12.1%) of peripheral blood samples. Diagnostic efficacy of mNGS was similar between single blood samples and multiple specimen types in febrile patients but superior when combined specimens were used in patients with additional symptoms.

Achieving such quick results requires specialized sequencing equipment, optimized laboratory workflows, and personnel with expertise in bioinformatics to analyze the complex data. Without these resources, mNGS processing may take longer, and accurate interpretation of findings can be challenging [26]. Early identification yields fast, targeted treatment that lowers empirical broad-spectrum antibiotic use and improves antimicrobial stewardship.

Although mNGS offers many advantages, several challenges and limitations restrict its clinical value. A major challenge is the possibility for false-positive results, usually resulting from environmental contaminants or normal skin flora introduced during sample collecting or processing. These can complicate result interpretation especially in sterile-site infections. The detection rate of false-positive pathogens by mNGS was significantly higher than that of conventional methods, highlighting the need to carefully differentiate true positives from false positives when interpreting mNGS results [27]. The increased detection rate of false-positive pathogens by mNGS underscores the critical role of clinical judgment in differentiating incidental findings from true infections. While mNGS provides unparalleled sensitivity, its ability to detect low-biomass or background microorganisms can complicate interpretation, particularly in complex clinical cases. Moreover, mNGS's financial cost and limited availability could make regular adoption challenging, especially in resource-limited healthcare settings [28].

Direct comparisons are challenging due to variability in clinical settings, study designs, and sample types (eg, tissue, pus, cerebrospinal fluid). Some studies lacked appropriate control groups or included small sample sizes in key subgroups, such as fungal infections which may skew results [31]. Additionally, outcome measures and reporting, including treatment changes and time to diagnosis, were inconsistent across studies. Nevertheless, the consistent performance of mNGS across diverse settings supports its reliability as a diagnostic tool.

These findings support the inclusion of mNGS into diagnostic routes for spinal infections, particularly in challenging conditions including culture-negative cases, atypical clinical presentations, or those with past antibiotic exposure that may suppress pathogen growth in conventional cultures. Nonetheless, mNGS should be seen as a complementary tool to improve diagnostic accuracy rather than as a substitute for conventional microbiological techniques [29].

Based on clinical evaluation and imaging results, a pragmatic clinical approach might call for starting empirical antimicrobial treatment and then using mNGS to confirm diagnosis or modify treatment plans depending on pathogen identification [30]. Prospective studies with standardized diagnostic criteria should take precedence to confirm the value of mNGS in practical environments. Furthermore, crucial for determining the viability of general acceptance are cost-effectiveness studies [31]. Combining host-response biomarkers with mNGS might improve diagnostic specificity and help to differentiate actual infection from colonization or contamination. The actual advantages of mNGS-guided therapy approach ultimately depend on robust clinical outcome measures including mortality, length of hospital stay, and rates of infection resolution.

### Limitations

This study revealed several restrictions that might compromise the generalizability and interpretability of the findings. One common constraint was the small sample size, especially within specific subgroups, which lowered the statistical power of the studies. Many studies applied retroactive designs, so compromising control over confounding factors and raising risk of selection bias. Moreover, frequently seen was past antibiotic use, which most likely lowered cultural positivity rates and may have influenced the apparent superiority of mNGS. Lack of a consistent diagnostic gold standard across studies added still another major challenge, making direct comparisons and result validation challenging. In the end, inconsistent or inadequate reporting of clinical treatment outcomes degraded the ability to fully assess mNGS-guided treatments on patient care.

## Conclusion

This systematic review and meta-analysis set out to answer 5 key clinical questions regarding the diagnostic and therapeutic utility of mNGS in native pyogenic spinal infections. These findings support the integration of mNGS into standard diagnostic pathways for spinal infections as a powerful complement to traditional methods, particularly in complex or culture-negative cases.

## Declaration of competing interest

The authors declare that they have no known competing financial interests or personal relationships that could have appeared to influence the work reported in this paper.

## Appendix Supplementary materials

Image, application 1
