# Supplementary material for: The diagnostic value of metagenomic next-generation sequencing versus traditional microbiological testing in native pyogenic spinal infections: A systematic review and meta-analysis
Source: N Am Spine Soc J. 2025 Dec 20;25:100840. doi: 10.1016/j.xnsj.2025.100840 (PMC12830174; doi:10.1016/j.xnsj.2025.100840)
Supplement: Supplementary file 1 [file mmc1.docx]

**Supplementary Table 1: Data extraction of the included studies**

| **Study No.** | **Authors** | **Objective** | **Study type** | **Participants** | **Gender distribution (Male)** | **Gender distribution (Female)** | **Age (years)** | **Clinical conditions** | **Sensitivity (mNGS)** | **Specificity (mNGS)** | **Positive Predictive Value** | **Negative Predictive Value** | **Antimicrobial management** | **Diagnostic yield (% positive identification by mNGS)** | **Time to diagnosis** | **Treatment modification based on mNGS findings (what modification made)** | **Incidence of false positives/contamination (n, %)** | **Spectrum of pathogens detected by mNGS but missed by culture** | **Limitations of the study** | **Conclusion** |
| --- | --- | --- | --- | --- | --- | --- | --- | --- | --- | --- | --- | --- | --- | --- | --- | --- | --- | --- | --- | --- |
| 1 | Li et al. [12] | To evaluate clinical characteristics, diagnostic performance of mNGS, and outcomes in NB patients | Retrospective study | 20 | 13 | 7 | Median: 42 (IQR: 30–52) | Neurobrucellosis (NB) | 90% | NR | NR | NR | All patients received anti-brucellosis therapy | 90% | 2 weeks to 1 year (average 4.5 months) | Implied but not explicitly detailed | 1 potential false positive | Brucella detected by mNGS more often than culture | Retrospective design, small sample size, prior antibiotic treatment | mNGS is highly sensitive and valuable for early NB diagnosis and treatment decisions |
| 2 | Yin et al. [15] | To evaluate diagnostic performance of mNGS for spinal infection | Retrospective study | 120 | 80 | 40 | Infection group: 66.02 ± 11.81; Noninfection group: 59.24 ± 12.41 | Spinal infections and noninfectious spinal diseases | 86.44% | 92.00% | 96.23% | 74.19% | Antibiotic treatments guided by pathogen detection | mNGS positive rate: 88.42%; traditional culture: 43.16% | Within 48 hours | Treatment modified for rare pathogens | Minimal; rare detection of potential contaminants (e.g., Taifanglania major) | Coxiella burnetii, Taifanglania major detected by mNGS but missed by culture | Retrospective design, small number of some infection types, no gold-standard reference | mNGS is a rapid, sensitive, and valuable tool for diagnosing spinal infections and guiding treatment |
| 3 | Wang et al. [8] | To evaluate the diagnostic value of mNGS in acute spinal infections | Prospective observational | 114 | 61 | 53 | 62 ± 11.73 (infection group) | Definite acute spinal infection, clinical acute spinal infection, noninfectious diseases (e.g., tumor) | 97.83% | 100% | 100% | 80% | 34/82 (41.46%) changed antibiotics based on mNGS | 78.95% | NR | Empiric antibiotic changed immediately or after 4–7 days based on mNGS findings (switch to voriconazole, or add doxycycline + gentamicin) | Background microorganisms detected: Propionibacterium acnes and others | Viruses, mycoplasma, MTBC | Small sample size of control group, potential contamination, prior antibiotic use affecting culture | mNGS improves pathogen detection and guides targeted treatment, outperforming conventional methods |
| 4 | Li et al. [14] | To compare mNGS with microbial culture to explore the diagnostic value in pyogenic spinal infections | Multicenter, retrospective observational study | 301 | 186 | 115 | 61.63 ± 13.40 | Pyogenic spinal infections | 77.90% | NR | NR | NR | NR | 77.9% (mNGS), 27.2% (culture) | 24–48 hours (mNGS) vs 2–7 days (culture) | NR | NR | Pathogens detected by mNGS even in culture-negative cases (73.1%) | NR | mNGS serves as a valuable supplement to culture, improves detection and speeds up diagnosis |
| 5 | Zhang et al. [13] | To evaluate mNGS in spinal infections | Retrospective observational study | 38 | 29 | 9 | 57.4 ± 12.9 | Spinal infection (vertebral osteomyelitis, discitis, paravertebral infection, epidural abscess) | 84.20% | 100.00% | 100.00% | 40% | mNGS-guided antibiotic therapy | 78.95% (mNGS), 44.74% (culture) | 24–48 hours (mNGS) vs 2-7 days (culture) | Antibiotics were adjusted based on mNGS or culture results; therapy guided by pathogen identification | NR | M. tuberculosis complex, S. aureus, Brucella, M. hominis, S. agalactiae, and others | Small sample size, no significant difference in detection between tissue and pus samples | mNGS shows higher detection rates and faster results compared to microbial culture, aiding in accurate pathogen identification |
| 6 | Li et al. [9] | To compare mNGS with traditional methods for pathogen detection | Prospective, observational study | 27 | 12 | 15 | Median: 61.5 (Range: 40–83) | Tuberculous spondylitis, fungal infection, Brucella spondylitis, viral infection, bacterial infection, mixed infections | 85.19% vs. 48.15% | NR | NR | NR | No treatment modification mentioned | 85.19% | Less than 2 days (mNGS), 2–10 days (culture) | mNGS led to diagnosis of fungal spondylitis and bacterial infections missed by culture | 0 | mNGS detected a higher variety of pathogens compared to culture | Study limited by small sample size and non-uniformity in pathogen detection | mNGS shows higher diagnostic accuracy and shorter time to diagnosis compared to traditional methods |
| 7 | Xu et al. [17] | To evaluate the diagnostic performance of metagenomic next-generation sequencing (mNGS) for spinal infections. | Observational study | 108 | 55 | 53 | Median: 57.8 (Range: 14.0–82.0) | Lumbar spine (75.9%), thoracic spine (18.5%), cervical spine (0.9%) | mNGS: 90.72%, CMTs: 52.17% | mNGS: 81.82%, CMTs: 56.25% | NR | NR | mNGS-guided antimicrobial therapy | mNGS detected novel pathogens (15 bacteria, 5 NTM, 4 fungi) | 42.8 days (median) | Antibiotics were adjusted based on mNGS findings (specific pathogens identified) | Not reported, but contamination in cultures noted | Mycobacteria, E. coli, Staphylococcus spp., NTM, fungi | Low concentration of nucleic acid extraction; some CMTs were false positive due to contamination | mNGS was superior to CMTs in identifying pathogens, leading to better treatment outcomes, and improved patient conditions upon discharge. |
| 8 | Li et al. [18] | To evaluate the diagnostic performance of mNGS in spinal tuberculosis (STB) | Retrospective study | 126 | 76 | 50 | STB: 52.63 ± 18.82, non-STB: 53.92 ± 15.33 | Suspected spinal infections (STB and non-STB) | 39.00% | 98.80% | 94.10% | 77.10% | NR | 69.00% | NR | Treatment adjustments made based on mNGS findings not detailed explicitly | NR | Pathogens detected by mNGS included MTB and other organisms; mNGS detected pathogens missed by culture in several cases | Retrospective design; no detailed report on treatment outcomes based on mNGS; small sample size for subgroup analysis | mNGS improves pathogen detection in spinal infections but has moderate sensitivity for STB compared to traditional methods; combining assays improves diagnostic performance |
| 9 | Li et al. [10] | To evaluate performance of mNGS in diagnosing spinal infections | Prospective study | 100 | 52 | 48 | Mean: 47.7 ± 16.5 | Spinal TB, non-TB spinal infection, non-infectious spinal diseases | 89.0% overall; 94.7% in spinal TB; 84.9% in non-TB infection | 88.9% overall; 100% in spinal TB; 97.9% in non-TB infection | 98.8% overall; 100% in spinal TB; 97.8% in non-TB infection | 44.4% overall; 96.9% in spinal TB; 85.2% in non-TB infection | NR | 82% | Mean: 17.7 ± 1.7 hours | NR | NR | Brucella, Staphylococcus aureus, Escherichia coli, fungi (Candida species), Streptococcus anginosus, Klebsiella pneumoniae | Single-center study, small non-infectious control group, need for larger multicenter validation | mNGS showed high sensitivity and specificity, especially valuable in spinal infections; useful for early diagnosis and guiding therapy |
| 10 | Ali et al. [16] | To evaluate the diagnostic performance of mNGS vs CMTs in infection detection | Comparative observational study | 85 | 49 | 36 | 54.52 ± 19.24 | CHD, DM, COPD | 92.06% | 54.55% | 85.29% | 70.59% | Cephalosporins, piperacillin sodium, vancomycin, combination therapy, moxifloxacin | Infection group had significantly higher positive mNGS results than non-infection group (P < 0.0001) | NR | Not explicitly described | 5 discordant results between NGS and CMTs (incidence not given in %) | mNGS detected additional pathogens compared to CMTs (specific pathogens not listed) | Slight agreement between NGS and CMTs (κ = 0.062); no significant association (P = 0.1488); relatively low specificity | mNGS showed superior sensitivity and NPV compared to CMTs, making it more effective for detecting true infections |
| 11 | Zhang et al. [11] | To evaluate the value of mNGS in the diagnosis and management of spinal infections | Prospective observational study | 158 | 81 | 77 | 54 | Spinal infections | 86.96% | 16.20% | 100% | 16.20% | 80 patients received targeted therapy based on mNGS | 86.96% | Faster than culture (exact time not stated) | Switch from empirical to targeted antimicrobial therapy | NR | Mycobacterium tuberculosis, fungi, viruses, mixed infections | Single-center study; limited sample size; low culture positivity; lack of full cost-effectiveness analysis | mNGS significantly improves the diagnostic yield for spinal infections compared to traditional methods and facilitates targeted treatment |
| 12 | Ma et al. [19] | To evaluate the accuracy of mNGS in determining the etiology of spinal infection | Retrospective study | 30 | 16 | 14 | 63.0±9.0 | Suspected spinal infection (infected: 26, aseptic: 4) | 70.3% | 75.0% | 95.0% | 27.3% | Not detailed; implication of guided therapy in TB and mixed infections | 73.1% | NR | mNGS guided diagnosis and effective anti-TB therapy in mixed infection; implication of modifying treatment based on detected pathogens | 1/4 aseptic cases (25%) = contamination (S. epidermidis) | Pseudomonas aeruginosa, *Escherichia coli, K. variicola, Candida albicans* (in mixed infections, not detected by culture) | Small sample size, retrospective design, absence of quantitative mNGS analysis, contamination risk | mNGS is a promising adjunctive tool for pathogen identification in spinal infections, especially when culture fails. |
